# *Vangueria madagascariensis* Fruit Tree: Nutritional, Phytochemical, Pharmacological, and Primary Health Care Applications as Herbal Medicine

**DOI:** 10.1155/2018/4596450

**Published:** 2018-11-01

**Authors:** Alfred Maroyi

**Affiliations:** Medicinal Plants and Economic Development (MPED) Research Centre, Department of Botany, University of Fort Hare, Private Bag X1314, Alice 5700, South Africa

## Abstract

*Vangueria madagascariensis* J. F. Gmel. is a plant species regarded as an important fruit tree and medicinal plant in sub-Saharan Africa. This study critically reviewed the nutritional value, phytochemistry, medicinal uses, and pharmacological properties of *V. madagascariensis*. Relevant information on food and medicinal uses of the species was collected from electronic databases such as ISI Web of Knowledge, ProQuest, ScienceDirect, OATD, Scopus, OpenThesis, PubMed, and Google Scholar, and preelectronic literatures were obtained from the university library covering the period 1966 to 2018. Literature studies revealed that *V. madagascariensis* has been integrated into farming systems as a fruit tree to support income and nutritional security of households in the region. *Vangueria madagascariensis* is used as a herbal medicine against diabetes, gastrointestinal problems, malaria, pain, parasitic worms, and skin diseases. Phytochemical compounds identified from the species include alcohols, aldehydes, esters, furanoids, ketones, and terpenoids. Pharmacological studies revealed that *V. madagascariensis* extracts have antibacterial, anticonvulsant, antidiabetic, antifungal, anti-inﬂammatory, antioxidant, cytotoxicity, antimalarial, and antiplasmodial properties. *Vangueria madagascariensis* should be subjected to detailed nutritional, pharmacological, and toxicological evaluations aimed at correlating the traditional uses of the species and the scientific evidence as well as establishing the efficacy, clinical relevance, safety, and mechanisms of action of the plant extracts and compounds.

## 1. Introduction

**Figure 1:**
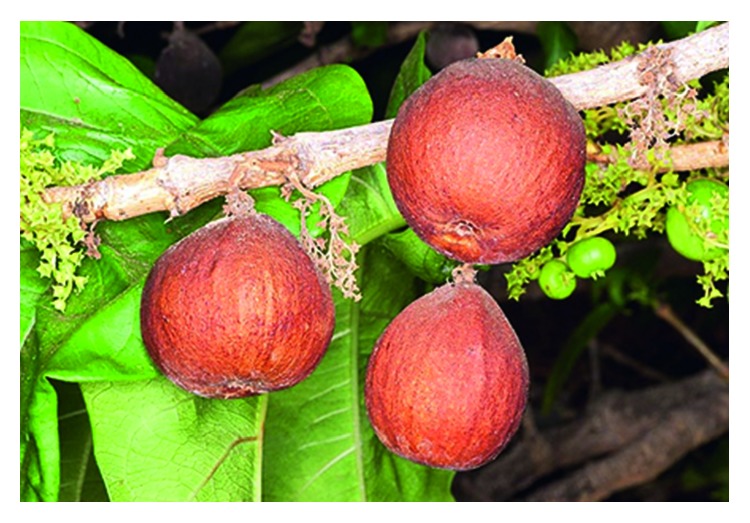
*Vangueria madagascariensis*: a branch showing leaves, flowers, and fruits (photo: Guiseppe Mazza).

Over the last three decades, there has been renewed interest in the phytochemical properties of *Vangueria madagascariensis* J. F. Gmel. (Figure 1), a plant species with edible fruits and used as a herbal medicine throughout its distributional range. *Vangueria madagascariensis* belongs to the bedstraw or family Rubiaceae, which is regarded as one of the largest plant groups characterized by about 637 genera and 13000 taxa [1, 2]. *Vangueria madagascariensis* is a type of species of the genus *Vangueria* Juss., which formed a strongly supported group or clade including *V. esculenta* S. Moore, *V. infausta* Burch., and *V. proschii* Briq. based on the results of chloroplast markers trnT-F and rps16 and nuclear ITS [1]. All these four species are characterized by calyx lobes that are narrow, oblong, and triangular in shape [1]. *Vangueria* genus is made up of about 50 small trees, shrubs, and geofrutices species distributed in sub-Saharan Africa with *V. madagascariensis* also occurring in Madagascar, Mauritius, Réunion, and Seychelles [1, 3]. East Africa, particularly Kenya and Tanzania, is regarded as the centre of diversity of this genus, which is regarded as rare in West Africa [1]. The genus name *Vangueria* is based on the local Malagasy name “voa vanguer” of *V. madagascariensis* [4–6]. The specific name “*madagascariensis*” means “of Madagascar” in reference to Madagascar where the specimen type was collected from the species described in 1791 by Johann Friedrich Gmelin (1748–1804), a German naturalist and botanist. English common names of *V. madagascariensis* include common wild medlar, Spanish-tamarind, tamarind-of-the-Indies, voa vanga, and wild medlar [7–9]. The synonyms of the species include *V. acutiloba* Robyns, *V. edulis* Vahl., *V. floribunda* Robyns, *V. madagascariensis* subsp. *madagascariensis*, *V. madagascariensis* var. *madagascariensis*, *V. robynsii* Tennant, *V. venosa* Hochst. ex A. Rich., *Vavanga chinensis* Rohr, and *Vavanga edulis* Vahl [3].

**Figure 2:**
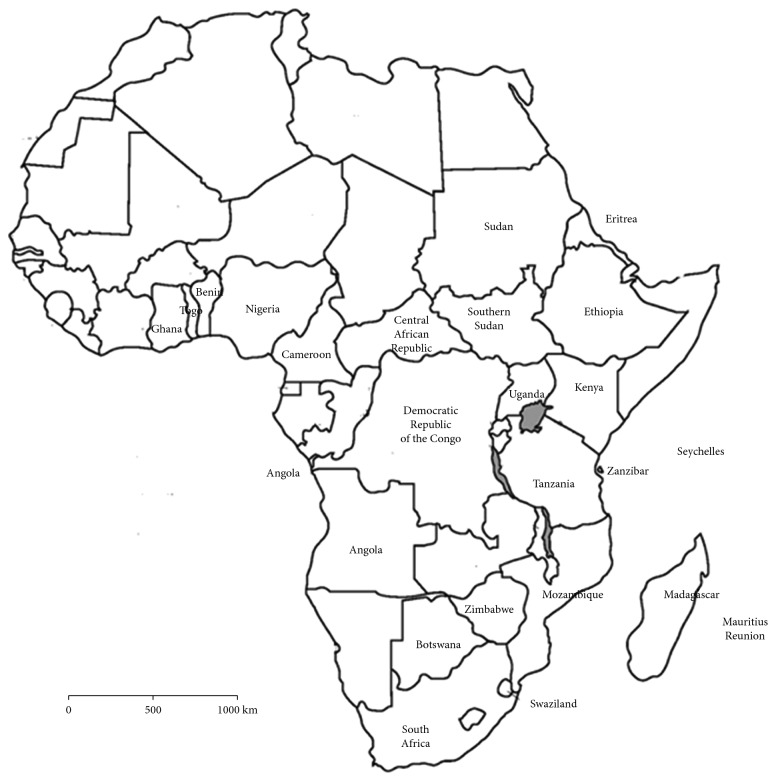
Natural distribution of *Vangueria madagascariensis*.

*Vangueria madagascariensis* is a multistemmed deciduous shrub or tree growing up to 15 metres in height. The species is native to Réunion, Tanzania, Democratic Republic of Congo, South Sudan, Angola, Cameroon, Ghana, Benin, Central African Republic, Ethiopia, Eritrea, Madagascar, Mauritius, Mozambique, Nigeria, South Africa, Sudan, Swaziland, Togo, Uganda, Seychelles, and Kenya [3] (Figure 2). The species has been recorded on rocky, sandy red clay, or sandy clay soils in riverine bushlands, evergreen forests, bushed grasslands, rocky outcrops, and termite mounds at an altitude ranging from 0 to 2400 metres above the sea level [3]. *Vangueria madagascariensis* is cultivated in China, Congo, Cuba, India, northern Australia, Singapore, and Trinidad [8–12]. Considering the existing literature focusing on utilization of *V. madagascariensis* throughout its distributional range, it is evident that different plant parts are used as both food and herbal medicines as these plant parts have several phytochemical compounds and micronutrients required for human nutrition and health [9, 13–17]. Previous research showed that the medicinal and nutritional properties of edible fruits collected from the wild enable local communities to use such plant resources as traditional remedies, at the same time broadening their nutritional options, micronutrients, diet, and vitamins [6, 18, 19]. *Vangueria madagascariensis* is regarded as a popular fruit tree and medicinal plant, and the plant species has positive effects on human health and well-being [9, 13–17] which are beyond the provisions of basic nutritional requirements. There is no universally accepted definition of functional food and nutraceuticals, but Hailu et al. [20], Shahidi [21], and Wang and Li [22] argued that functional food and nutraceuticals are natural foods that beneficially affect one or several body functions apart from nutritional effects, influencing both the health and well-being of the consumer. The value of pharmaceutical drugs derived from plants, other natural health products, nutraceuticals, and functional foods are being promoted throughout the world as an alternative strategy for disease risk reduction and reduction in health care costs [21]. It is within this background that the nutritional value, chemical properties, medicinal uses, and biological activities of *V. madagascariensis* were evaluated.

## 2. Food Uses

The fruits of *V. madagascariensis* which are globose, smooth and shiny, and yellowish brown in colour when ripe are edible and highly sought after throughout the distributional range of the species. Although the fruits are mainly collected from the wild, but in clearing land for agricultural purposes, some farmers leave these trees as future sources of fruits. Teklehaimanot [23] identified *V. madagascariensis*, *Strychnos cocculoides* Baker, *Balanites aegyptiaca* (L.) Delile, *Vitex doniana* Sweet, *Berchemia discolor* (Klotzsch) Hemsl., *Sclerocarya birrea* (A. Rich.) Hochst., *Borassus aethiopum* Mart., *Carissa spinarum* L., *Cordeauxia edulis* Hemsl., and *Vitellaria paradoxa* C. F. Gaertn. as priority indigenous fruit tree species with domestication potential in Ethiopia, Kenya, South Sudan, Sudan, Tanzania, and Uganda. Therefore, *V. madagascariensis* has been integrated into rural agricultural farming systems in sub-Saharan countries as a strategy to improve food and income security of households in the region. Based on the popularity of its fruits, *V. madagascariensis* was also introduced in home gardens in Congo, Cuba, India, and the West Indies [8–10]. The fruits are marketed in Cuba, Ethiopia, Kenya, Madagascar, Tanzania, and Uganda [8–10, 24, 25].

The fruit pulp of *V. madagascariensis* has a sweet, pleasant chocolate-like flavour when eaten raw and a somewhat astringent and acidic taste like a blend of apple (*Malus pumila* Miller) and tamarind (*Tamarindus indica* L.) [8, 26]. The pulp is also stewed, roasted, added to mealie meal porridge and other food to add flavour, and made into juice, jellies, jam, and puddings [26–29]. In Ethiopia, fruits of *V. madagascariensis* are an important food resource especially during droughts and in times of food shortages [30]. The pulp is a good source of both macrominerals and trace elements such as potassium, zinc, calcium, magnesium, chromium, phosphorus, copper, manganese, and iron (Table 1). The nutritional contribution of the pulp is comparable to other well-known fruits with commercial potential such as *Mangifera indica* L. and *Ziziphus mauritiana* Lam (Table 1). *Mangifera indica* and *Ziziphus mauritiana* are among the top five important fruit species in the dryland agricultural farming systems in tropical Africa that contribute to household incomes, nutritional needs, and food security [38]. Several amino acids and fatty acids (Table 2) have been identiﬁed from the fruit pulp of *V. madagascariensis*, and these include the essential amino acids such as lysine, threonine, histidine, leucine, phenylalanine, valine, isoleucine, and methionine [39, 44]. Research by Mariod et al. [15] revealed that the contribution of conditionally essential amino acids such as tyrosine, arginine, glycine, and cysteine and nonessential amino acids such as glutamic acid, aspartic acid, and serine was close to 50% (5.9 g out of 14.2 g/100 g) of the total amino acids identified from the species (Table 2). The amino acid and fatty acid constituents and other physicochemical properties of *V. madagascariensis* make the species a valuable source of these nutrients when compared with the nutritional value of *Mangifera indica* and *Ziziphus mauritiana* and the FAO/WHO/UNU dietary reference intakes or RDA required to meet essential nutrients for a healthy person (Tables 1 and 2). Pino et al. [9] identified sixty volatile constituents from the fruit pulp of *V. madagascariensis* (Table 3). The major phytochemical compounds identified include alcohols, aldehydes, esters, furanoids, ketones, and terpenoids (Table 3). Pino et al. [9] argued that the acidic and pungent taste associated with the fruit pulp of *V. madagascariensis* can be explained by the higher amounts of fatty acids as shown in Table 2.

## 3. Medicinal Uses of *Vangueria madagascariensis*

The seeds, bark, leaves, fruits, roots, and stem bark of *V. madagascariensis* are utilized in monotherapeutic or multitherapeutic applications in Eritrea, Kenya, Madagascar, Mauritius, Sudan, and Tanzania (Table 4). Bark, fruit, leaf, and root maceration of *V. madagascariensis* is taken by mouth for diabetes in Madagascar [47], Mauritius [14, 48], and Sudan [49, 50]. Bark, leaf, root bark, and stem bark infusion of *V. madagascariensis* is taken by mouth for bloody diarrhoea in Tanzania [45], dysentery in Mauritius [47], and stomach problems in Kenya [58]. Root bark and root infusion of *V. madagascariensis* is taken by mouth for intestinal worms in Eritrea [52] and Tanzania [8, 45]. Bark, root bark, and stem bark maceration of *V. madagascariensis* is taken by mouth for malaria in Kenya [53–57] and Tanzania [8, 45, 51]. In Tanzania, the leaf, root, root bark, and stem bark maceration of *V. madagascariensis* is taken by mouth for abdominal pains, asthma, convulsions, gonorrhoea, hepatitis, hernia, and oedema [45, 51]. Fruit decoction of *V. madagascariensis* is taken by mouth for back pain and mouth infections in Kenya [46, 58], while root decoction is taken orally as a purgative in Eritrea [52]. In Sudan, the fruit and seed decoction of *V. madagascariensis* is taken orally as a remedy for hypertension, kidney problems, and tumour [50, 59], while bark and leaf decoction is taken orally for palpitations and nausea in Mauritius [47]. Multitherapeutic applications of *V. madagascariensis* involve mixing leaves of the species with leaves of *Jatropha curcas* L., *Azadirachta indica* A. Juss., and *Ipomoea pes-caprae* (L.) R. Br. as a herbal medicine for abscesses, carbuncle, and scurf in Mauritius [14]. In Mauritius, the leaf decoction of *V. madagascariensis* is mixed with the leaves of *Jatropha curcas*, *Toddalia asiatica* (L.) Lam., and *Sporobolus africanus* (Poir.) Robyns & Tournay as a mouthwash [48].

## 4. Phytochemistry and Pharmacological Properties of *Vangueria madagascariensis*

**Figure 3:**
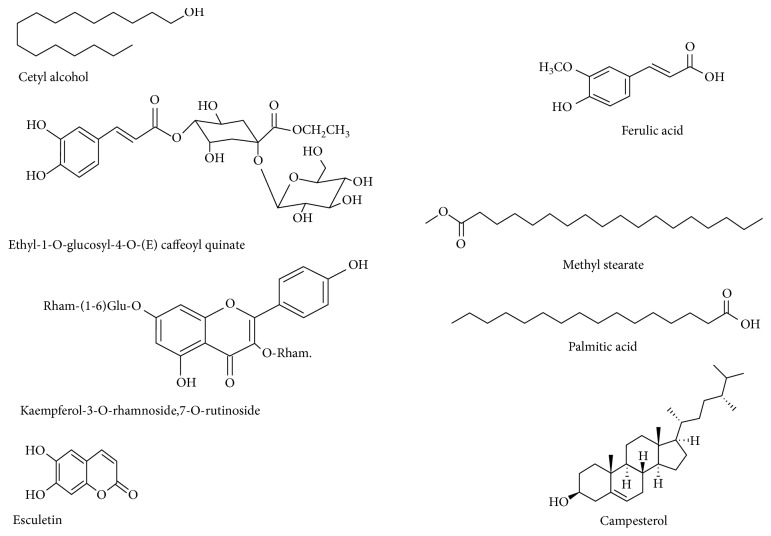
Phytochemical structures of representative phytochemical compounds isolated from *Vangueria madagascariensis*.

Phytochemical screening of the bark, fruits, leaves, kernel oil, seeds, stems, and stem bark has shown the presence of fibre, carbohydrates, proteins, and several classes of phytochemicals such as volatile and nonvolatile metabolites (Table 5), and chemical structures of representative phytochemical compounds are shown in Figure 3. The majority of the phytochemicals were identified using high-performance liquid chromatography (HPLC-DAD) with diode array detection (DAD), mass spectrometry (MS), gas chromatography-mass spectrometry (GC/MS), nuclear magnetic resonance (NMR) spectroscopy, and gas chromatography (GC) (Table 5). Each fruit of *V. madagascariensis* has 4 to 5 seeds, and the seed kernel contains considerable amount of oil which is higher than that of conventional oil seeds such as groundnut (*Arachis hypogaea* L.), cottonseed (*Gossypium hirsutum* L.), and sunflower (*Helianthus annuus* L.) [62]. Some of the phytochemical compounds such as flavonoids identified from *V. madagascariensis* are known to have antiallergic, anti-inflammatory, antimicrobial, antiproliferative, antioxidant, enzyme inhibition, and oestrogenic activities, synergism with antibiotics, and suppression of bacterial virulence [63–66]. Research by Prochazkova et al. [65] revealed that the antioxidant activities of flavonoids involve quenching free radical elements, metal chelation, suppression of enzymes involved in free radical scavenging, and stimulation of enzymes that activate antioxidant activities. Research has also revealed that food resources characterized by high levels of flavonoids and related phenolic compounds may reduce the risk of cardiovascular diseases [67]. Pereira et al. [63] argued that the structural figure of phenolic compounds has the potential to interact with several proteins; mainly, they have a hydrophobic benzenoid ring and hydrogen-binding properties which enhance their capacity to be antioxidants by inhibiting several enzymes that catalyze radical generation, including xanthine oxidase, cytochrome P450 isoforms, cyclooxygenase, and lipoxygenase enzymes. The different parts of *V. madagascariensis* are associated with several fatty acids [16, 60, 61], and these compounds are known to have a wide range of physiological effects such as cardiovascular function, immune system regulation, neuronal development, regulation of plasma lipid levels, insulin regulation, and visual function [68]. Several studies done elsewhere demonstrated the importance of dietary intake of fatty acids as they lead to reduced blood pressure, they lower the risk of heart attack and arteriosclerosis risks, and these compounds are associated with antimicrobial properties and synthetic accessibilities [69–71]. Desbois and Smith [71] argued that the antimicrobial properties of fatty acids are based on their ability to disturb and distort the oxidative phosphorylation process and the electron transport chain process, thereby disturbing the cellular energy production, leading to reduction of enzymatic activity, reduced nutrient uptake, and production of toxic peroxidation. The phytochemicals detected in various parts of *V. madagascariensis* may be used to justify some of the medicinal uses of this species recorded in Table 4 and also documented antibacterial [13, 60, 72], anticonvulsant [60], antidiabetic [13, 73], antifungal [60, 74], anti-inﬂammatory [60], antioxidant [13, 17], cytotoxicity [17], antimalarial, and antiplasmodial [55, 56] activities.

### 4.1. Antibacterial Activities

Bishay et al. [60] assessed antibacterial properties of leaf and bark ethyl acetate, chloroform, and *n*-hexane extracts of *V. madagascariensis* against *Pseudomonas aeruginosa*, *Bacillus cereus*, *Escherichia coli*, *Klebsiella pneumoniae*, *Micrococcus luteus*, and *Staphylococcus aureus* using the modified diffusion method with dimethylformamide (DMF) and gentamicin (5 *µ*g/ml) as negative and positive controls, respectively (Table 6). The extracts showed activities with the zone of inhibition stretching from 4 mm to 18 mm which was comparable to the zone of inhibition of 10 mm to 14 mm demonstrated by gentamicin, the control. The minimum inhibitory concentration (MIC) values ranged from 6.3 *µ*g/ml to 75 *µ*g/ml [60]. Ramalingum and Mahomoodally [13] evaluated antibacterial properties of methanol and crude fruit, leaf, and seed extracts of *V. madagascariensis* making use of the disc diffusion and microtitre dilution broth method against *Escherichia coli* and *Staphylococcus aureus* with streptomycin sulphate and gentamicin sulphate as positive controls (Table 6). The crude ripe and unripe fruit extracts and methanol leaf and seed extracts exhibited some antibacterial properties with the zone of inhibition ranging 8.3 mm to 12.7 mm and MIC values ranging from 6.3 mg/mL to 25.0 mg/mL [13]. Mahomoodally and Dilmohamed [72] evaluated antibacterial activities of fruit and leaf extracts of *V. madagascariensis* against *Klebsiella* spp., *Acinetobacter* spp., *Enterococcus faecalis*, *Pseudomonas aeruginosa*, *Staphylococcus aureus*, *Proteus* spp., *Streptococcus* spp. and methicillin-resistant *Staphylococcus aureus* (MRSA), and *Escherichia coli* using the microdilution broth method with chloramphenicol and gentamicin as positive controls (Table 6). The extracts demonstrated antibacterial properties with MIC values ranging from <0.1 mg/mL to 12.5 mg/mL. The authors found that mixing of antiobiotics such as chloramphenicol and gentamicin with *V. madagascariensis* extracts resulted in significant antibacterial properties by reducing the MICs [72]. These antibacterial activities exhibited by different extracts of *V. madagascariensis* corroborate the traditional use of the species as herbal concoction against bacterial and other microbial infections causing bloody diarrhoea and gonorrhoea in Tanzania [45], carbuncle and dysentery in Mauritius [14, 48], mouth infections in Kenya and Mauritius [48, 58], and stomach problems in Kenya [58].

### 4.2. Anticonvulsant Activities

The activities of the different fractions and the total ethanolic extracts of both leaves and stem bark of *V. madagascariensis* on the central nervous system were evaluated by performing assays of their effect on motor coordination (rotarod test) and pentylene tetrazole-induced convulsion [60]. The total ethanolic extracts as well as the other fractions of both leaves and stem-bark attained a central nervous system depressant activity. The n-butanol and n-hexane extracts of the leaves, n-hexane and chloroform extracts the stem-bark at 400 mg/kg have anticonvulsant properties against pentylene tetrazole induced convulsions in comparison with carbamazepine. The n-butanol and n-hexane extracts of the leaves, n-hexane and chloroform extracts of the stem-bark at 400 mg/kg exhibited anticonvulsant properties against pentylene tetrazole induced convulsions in rats in comparison with carbamazepine [60]. The anticonvulsant properties demonstrated by the extracts of *V. madagascariensis* corroborate the usage of stem bark of the species as herbal concoction against convulsions in Tanzania [60].

### 4.3. Antidiabetic Activities

Ramalingum and Mahomoodally [14] evaluated antidiabetic activities of methanol and crude fruit, leaf and seed extracts of *V. madagascariensis* using *α*-amylase, *α*-glucosidase and a modified glucose based colorimetric and glucose movement assays with acarbose as the control (Table 6). The crude and methanol extracts exhibited inhibitory activities against *α*-amylase with half maximal inhibitory concentration (IC_50_) values varying from 1.1 mg/mL to 29.6 mg/mL which was comparable to acarbose with IC_50_ value of 0.1 mg/mL. The extracts that exhibited activities against *α*-glucosidase where unripe fruit decoction, ripe fruit methanol, unripe fruit methanol, leaf decoction exhibited IC_50_ values stretching from 0.4 mg/mL to 3.3 mg/mL which were significantly lower than IC_50_ value of 5.0 mg/mL demonstrated by the control, acarbose. The kinetic evaluations showed a mixed non-competitive type of inhibition. Beidokhti et al. [73] evaluated the inhibition of pancreatic *α*-amylase and yeast *α*-glucosidase by ethanolic bark and leaf extracts of *V. madagascariensis* (Table 6). The showed activities against both *α*-glucosidase and *α*-amylase and characterized by IC_50_ values of 1.8 *µ*g/mL and 11.6 *µ*g/mL, respectively [73]. The observed antidiabetic activities of *V. madagascariensis* extracts support the use of the bark, leaves, fruits and roots of the species as herbal medicine against diabetes in Madagascar, Mauritius and Sudan [14, 47–50].

### 4.4. Antifungal Activities

Bishay et al. [60] assessed antifungal properties of bark and leaf aqueous, n-hexane, chloroform, ethyl acetate extracts of *V. madagascariensis* against *Candida albicans* using the modified diffusion method with clotrimazole (5 *µ*g/ml) as the positive control (Table 6). The bark and leaf extracts showed antifungal properties with zone of inhibition stretching from 9 mm to 20 mm which was comparable to zone of inhibition of 14 mm demonstrated by clotrimazole, the control. The MIC values stretched from 13.0 *µ*g/ml to 55.0 *µ*g/ml and clotrimazole, the control exhibited MIC value of 4.0 *µ*g/ml [60]. Similarly, Karim et al. [74] assessed antifungal properties of chloroform fruit extract of *V. madagascariensis* against *Candida albicans* and *Aspergillus Niger* using agar diffusion assay with ampicillin as positive control (Table 6). The chloroform fruit extract showed activities with zone of inhibition stretching from 14 mm to 15 mm [74]. These antifungal activities displayed by the different extracts of *V. madagascariensis* demonstrate the potential of the species in the management of fungal and microbial infections.

### 4.5. Anti-inﬂammatory Activities

Bishay et al. [60] assessed anti-inﬂammatory properties of bark and leaf aqueous, ethyl acetate, chloroform and n-hexane extracts of *V. madagascariensis* using the carrageenan-induced rat paw oedema model. Potent anti-inflammatory activities were observed after 2 hrs and continued for 4 hrs with all the extracts [60]. These findings corroborate the traditional use of *V. madagascariensis* as herbal concoction for abdominal pains in Tanzania [45], back pain in Kenya [46] and other various inflammatory ailments and diseases including skin infections and body injury that may lead to cell damage and death.

### 4.6. Antioxidant Activities

Ramalingum and Mahomoodally [13] evaluated antioxidant properties of methanol and crude fruit, leaf and seed extracts of *V. madagascariensis* using DPPH (1, 1-Diphenyl-2-picrylhydrazyl) free radical scavenging, hypochlorus acid (HOCl) scavenging, ferric reducing antioxidant power (FRAP), hydroxyl (^·^OH) radical scavenging or deoxyribose, nitric oxide radical (NO) scavenging and iron chelating property assays (Table 6). The methanol leaf, unripe and ripe fruit extracts exhibited antioxidant properties with IC_50_ values stretching from 9.0 *µ*g/mL to 48.5 *µ*g/mL which was comparable to ascorbic acid with IC_50_ value of 0.001 *µ*g/mL. All the extracts exhibited activities in the reduction of Fe^4+^ to Fe^2+^, confirming antioxidant properties with leaf methanol being the most active and seed decoction being the least active. The methanol unripe fruit extract exhibited the highest HOCl scavenging properties with IC_50_ value of 223.0 *µ*g/mL which was comparable to that of ascorbic acid, the control which exhibited IC_50_ value of 46.0 *µ*g/mL. The methanol leaf, unripe and ripe fruit extracts exhibited ˙OH scavenging properties with IC_50_ values stretching from 0.1 *µ*g/mL to 0.3 *µ*g/mL which were lower than that of *α*-tocopherol, the control which exhibited IC_50_ value of 0.5 *µ*g/mL. The leaf and unripe fruit crude extracts as well as methanolic leaf, ripe and unripe fruit extracts exhibited NO scavenging properties with IC_50_ values stretching from 43.2 *µ*g/mL to 436.2 *µ*g/mL which were lower than that of ascorbic acid, the control which exhibited IC_50_ value of 546.5 *µ*g/mL. All the extracts exhibited considerable iron chelating properties with IC_50_ values stretching from 0.0009 *µ*g/mL to 2.5 *µ*g/mL which were comparable to the positive control EDTA which exhibited IC_50_ value of 0.001 *µ*g/mL [13]. Mustafa et al. [17] assessed antioxidant properties of bark, leaf and seed extracts of *V. madagascariensis* using DPPH radical scavenging assay and oxygen radical absorbance capacity (Table 6). The DPPH assay revealed activities of leaf, seed and bark extracts with IC_50_ values of 7.8, 31.3 and 62.5 *μ*g/ml, respectively, which were comparable to IC_50_ value of 3.1 *μ*g/ml exhibited by ascorbic acid, the control. The oxygen radical absorbance ability findings revealed that the leaf extract demonstrated higher levels of antioxidant properties of 72.7 *μ*M of trolox than the control, quercetin (5 *μ*g/ml) which showed activity of 59.0 *μ*M of trolox), while the bark and seed extracts showed activities of 47.1 *μ*M of trolox and 44.9 *μ*M of trolox, respectively [17]. The documented antioxidant activities of *V. madagascariensis* extracts are probably due to flavonoids, phenolics and proanthocyanidins detected in fruits, leaves and stems of the species [14, 17, 60].

### 4.7. Cytotoxicity Activities

Mustafa et al. [17] assessed cytotoxicity properties of the bark, leaf and seed extracts of *V. madagascariensis* using the MTT [3-(4, 5dimethylthiazole-2-yl)2, 5-diphenyltetrazolium bromide] assay using human breast carcinoma (MCF-7), non-small cell lung cancer (A549), prostate adenocarcinoma (PC-3), human hepatocellular carcinoma (HepG2), human colon adenocarcinoma (HT-29), normal hepatic (WRL-68) and normal lung fibro blast (WI-38T) cells (Table 6). Test agents induced cell cytotoxicity in a concentration dependent trend with half maximal effective concentration (EC_50_) values stretching from 22.8 *μ*g/mL to 64.7 *μ*g/mL [17]. These findings corroborate the traditional use of *V. madagascariensis* as herbal concoction for tumour in Sudan [59].

### 4.8. Antimalarial and Antiplasmodial Activities

Muthaura et al. [55] assessed *in vivo* antimalarial properties of water and methanol stem bark of *V. madagascariensis* against *Plasmodium berghei* strain ANKA using a four-day suppressive assay with chloroquine as positive control. The *in vivo* studies showed weak activities with chemo-suppression of parasitaemia in *Plasmodium berghei* infected mice of 26.0% to 39.0% which was much lower than 99.9% exhibited by chloroquine [55]. Similarly, Muthaura et al. [55] assessed antiplasmodial properties of water and methanol stem bark of *V. madagascariensis* against chloroquine sensitive (D6) and resistant (W2) *Plasmodium falciparum* clones using the [G-^3^H]hypoxanthine incorporation assay with artemisinin and chloroquine as positive controls (Table 6). The methanol extracts exhibited moderate and weak activities with IC_50_ values of 13.4 *µ*g/mL and 34.0 *µ*g/mL against D6 and W2 strains, respectively. These results were higher than IC_50_ values demonstrated by the reference drugs artemisinin (*D*6 = 0.9 ng/mL, *W*2 = 3.4 ng/mL) and chloroquine (*D*6 = 9.0 ng/mL, *W*2 = 31.3 ng/mL) [55]. In another study, Muthaura et al. [56] assessed antiplasmodial properties of water and methanol stem bark of *V. madagascariensis* against chloroquine sensitive (D6) and resistant (W2) *Plasmodium falciparum* clones using the (G-^3^H) hypoxanthine incorporation assay with artemisinin and chloroquine as positive controls (Table 6). The methanol extracts demonstrated moderate and weak activities with IC_50_ values of 13.3 *µ*g/mL and 33.9 *µ*g/mL against D6 and W2 strains, respectively. These results were higher than IC_50_ values demonstrated by the reference drugs artemisinin (*D*6 = 0.9 ng/mL, *W*2 = 3.4 ng/mL) and chloroquine (*D*6 = 9.0 ng/mL, *W*2 = 31.3 ng/mL) [56]. Therefore, *V. madagascariensis* extracts showed promising antimalarial and antiplasmodial activities and these findings corroborate the traditional usage of the bark, roots and stem bark of the species as remedies against malaria in Kenya and Tanzania [8, 45, 51, 53–57].

### 4.8. Toxicity Activities

Bishay et al. [60] evaluated oral acute toxicity of bark and leaf n-hexane, chloroform, ethyl acetate extracts of *V. madagascariensis* by administering doses of 10 mg/kg, 100 mg/kg and 1000 mg/kg i.p. to male albino rats (Table 6). The treated animals were monitored for 24 hrs for symptoms of toxicity such as writhing, loss of motor co-ordination, irritability, hypothermia, sedation followed by deep sleep and finally death. The median lethal dose (LD_50_) values of 3.8 g/kg for both leaf and stem-bark extracts appear to suggest that the extracts of the species are safe to use as herbal medicines [60]. Muthaura et al. [55] assessed the acute, subacute and chronic toxicity of *V. madagascariensis* stem bark extracts by oral administration in female Swiss mice. The behaviour of mice was observed for 1 hour, intermittently for 4 hours, 24 hours and 14 days noting for any signs of toxicity and the latency of death. The extracts did not cause any mortality or signs of toxicity at any dose level up to the highest dose tested of 5000 mg/kg [55]. Since *V. madagascariensis* is widely utilized as both food and herbal medicine, there is need to ascertain toxicological properties of the species using different plant parts against several cell lines using both *in vitro* toxicological assays and *in vivo* studies.

## 5. Conclusion

*Vangueria madagascariensis* is an important functional food and source of nutraceutical ingredients in tropical Africa. Significant breakthrough has been made in the last 30 years elucidating the nutritional, phytochemical and pharmacological properties of the species. However, there are still some research gaps regarding correlating the nutritional and phytochemical properties of the species with its food value and medicinal applications. Detailed studies on the phytochemistry, pharmacokinetics, *in vivo* and clinical research are required. Further research on the antinutritive, enzymatic and molecular effects of *V. madagascariensis* fruits and kernel oil on human health will be needed to motivate further interest in the use of these products as food sources, additives and health promoting products. Given the situation that *V. madagascariensis* is used as herbal medicine in combination with other plant species such as *Azadirachta indica*, *Ipomoea pes-caprae*, *Jatropha curcas*, *Sporobolus africanus* and *Toddalia asiatica*, there is need to investigate the possibility of synergetic effects of the combined extracts. Since *V. madagascariensis* is a valuable functional food and nutraceutical plant species in tropical Africa, there is need to establish the toxicity and/or any side effects that can arise when the species and its products are used as functional food and sources of nutraceutical ingredients and/or as herbal medicines.

## Figures and Tables

**Table 1:** Nutritional composition of the fruit pulp of *V. madagascariensis* compared with nutritional values of *Mangifera indica* and *Ziziphus mauritiana* and the recommended dietary allowance (RDA).

Caloric and nutritional composition	Value	*Ziziphus mauritiana*	*Mangifera indica*	Recommended dietary allowance (RDA)
Ascorbic acid (mg/100 g)	4.7	15.0–43.8	16.0–46.5	100–120
Calcium (mg/100 g)	25	160–254	14.0–30.6	1000–1300
Carbohydrates (%)	28	79.5–83.2	16.9–27.3	45–65
Copper (mg/100 g)	0.5 ± 0.2	0.7–1.5	0.1	1–3
Chromium (mg/100 g)	0.2 ± 0.1	0.1	0.01–0.02	0.02–0.2
Energy value (kJ/100 g)	498	1516–1575	74	2200
Fibre (%)	4.7	4.9–7.3	1.1–4.8	25–38
Iron (mg/100 g)	1.1–5.2	2.1–4.3	1.3–8.4	8–15
Lipid (%)	0.1	—	0.1	300
Magnesium (mg/100 g)	39	83–150	1.5–7.5	310–320
Manganese (mg/100 g)	2.4 ± 1.1	0.7–1.6	6.2–7.8	1–5
Niacin (mg/100 g)	0.61	0-7–0.9	0.6	40–70
Phosphorus (mg/100 g)	36.6	87–148	16	1250
Potassium (mg/100 g)	521	1865–2441	10.2–205	4700
Protein (%)	1.4	7.9–8.7	0.6	34
Riboflavin (mg/100 g)	0.04	0.02	0.6	3–10
Sodium (mg/100 g)	28	185–223	26–91.1	2300
Thiamine (mg/100 g)	0.05	0.03	0.05	6.1
Total flavonoid content (mg RE/g fresh weight)	8.00 to 8.20	8.4–22.0		1000
Total phenolic content (mg GAE/g dry weight)	37.00 to 61.22	172.1–309.5	652.6	2500
Total proanthocyanidins (mg CE/g fresh weight)	134.57 to 159.50	—	7.9	1000
Zinc (mg/100 g)	0.4 ± 0.2	0.6–0.9	0.04	8–11

Sources: Ramalingum and Mahomoodally [14]; Nigam et al. [31]; Kipkemboi [32]; Nyanga et al. [33]; Pareek et al. [34]; Ara et al. [35]; da Silva et al. [36]; Sajib et al. [37].

**Table 2:** Fatty acids and amino acid composition of fruit pulp of *V. madagascariensis* compared with nutritional values of *Mangifera indica* and *Ziziphus mauritiana* and the recommended dietary allowance (RDA).

Chemical composition	Value	*Ziziphus mauritiana*	*Mangifera indica*	Recommended dietary allowance (RDA)
*Amino acids* (g*/100 *g)				
Arginine	1.1 ± 0.6	0.7	0.02	—
Aspartic acid	1.5 ± 0.7	1.3	0.04	—
Glutamic acid	1.9 ± 0.6	1.3	0.06	—
Glycine	0.8 ± 0.1	0.3	0.02	—
Histidine	0.7 ± 0.6	0.1	0.01	10
Isoleucine	0.82 ± 0.5	0.3	0.02	20
Leucine	1.6 ± 0.6	0.5	0.03	39
Lysine	0.8 ± 0.4	0.3	0.04	30
Methionine + cysteine	0.21 ± 0.1	0.1	0.01	15
Phenylalanine + tyrosine	1.3 ± 0.6	0.3	0.02	25
Serine	0.7 ± 0.4	0.3	0.02	—
Threonine	0.74 ± 0.4	0.3	0.02	15
Valine	1.0 ± 0.5	0.4	0.03	26

*Fatty acids* (*mg/kg*)				
Acetic acid	0.12	—	—	—
Butyric acid	0.12	—	—	—
Decanoic acid	0.08	—	—	—
Dodecanoic acid	0.30	0.05	0.02–0.5	—
Heptanoic acid	1.70	—	0.04–0.2	—
Hexadecanoic acid	5.19	4.0	2.2–14.6	—
Hexanoic acid	1.80	—	—	—
Octanoic acid	1.95	—	—	—
Octadecanoic acid	0.69	2.2	1.6–3.4	—
Pentadecanoic acid	0.61	—	0.02–0.04	—
Pentanoic acid	0.01	—	—	—
Undecanoic acid	0.04	—	—	—
Tetradecanoic acid	4.50	0.1	0.1–1.1	—
(Z)-9-Octadecenoic acid	0.06	—	—	—

Sources: Pino et al. [9]; Mariod et al. [15]; FAO/WHO/UNU [38]; Institute of Medicine [39]; Sena et al. [40]; Bally [41]; Vilela et al. [42]; Deshpande et al. [43].

**Table 3:** Volatile phytochemical compounds identified from *V. madagascariensis* fruits.

Phytochemical composition	Values (mg/kg)
*Alcohol*	
*α*-Terpineol	0.10
2-Methyl-3-buten-2-ol	1.07
Benzyl alcohol	0.25
Ethanol	0.08
2-Butanol	0.54
Isoamyl alcohol	1.38
2-Methylbutanol	0.24
3-Methyl-2-butenol	0.12
Octanol	0.51
Furfuryl alcohol	1.15
(Z)-3-Hexenol	0.06
Hexanol	2.40

*Aldehyde*	
2-Methylbutanal	0.43
2-Furfural	11.93
3-Furfural	2.43
2-Phenylacetaldehyde	2.12
Acetaldehyde	<0.01
Benzaldehyde	2.12
(E)-2-Octenal	3.84
(E,E)-2,6-Hexadienal	0.08
(E)-4-Undecenal	0.51
(E)-4-Nonenal	0.17
(E)-4-Decenal	0.09
(E,E)-4,4-Heptadienal	0.28
(E,Z)-4,4-Heptadienal	0.29
Heptanal	0.83
Hexanal	0.82
Isovaleraldehyde	0.44

*Ester*	
Methyl benzoate	0.56
Methyl 2-phenylacetate	0.34
2-Phenylethyl acetate	0.54
Methyl hexanoate	2.14
Methyl (Z)-3-hexenoate	<0.01
Methyl (E)-2-hexenoate	<0.01
Methyl octanoate	1.98
Methyl decanoate	0.05
Methyl butyrate	0.08
Methyl (E)-cinnamate	0.04
Methyl 9,12,15-octadecatrienoate	0.11
Methyl (Z)-9-hexadecenoate	0.51
Methyl hexadecanoate	0.39
Methyl octadecanoate	0.10
Methyl pentanoate	0.10
Methyl salicylate	0.08
Methyl tetradecanoate	<0.01

*Monoterpene*	
Terpinolene	0.09
p-Cymene	0.01
Limonene	2.48

*Furan*	
2-Propylfuran	<0.01
5-Methylfurfural	0.04

*Indole*	
1H-indole	0.04

*Ketone*	
2-Heptanone	0.28
2-Pentanone	0.41
3-Penten-2-one	0.10
Acetoin	0.04
5-Butyldihydro-2(3H)-furanone	<0.01
5-Ethyldihydro-2(3H)-furanone	1.95
*δ-*Octalactone	0.12
*γ*-Dodecalactone	0.04

*Norisoprenoids*	
4-Ketoisophorone	0.02

Source: Pino et al. [9].

**Table 4:** Medicinal applications of *Vangueria madagascariensis*.

Medicinal use	Parts of the plant used	Country	References
Abdominal pains	Roots	Tanzania	[45]
Abscesses, carbuncle, and scurf	Leaf decoction mixed with leaves of *Jatropha curcas* L., *Azadirachta indica* A. Juss., and *Ipomoea pes-caprae* (L.) R. Br.	Mauritius	[14]
Asthma	Leaves	Tanzania	[45]
Back pain	Fruits	Kenya	[46]
Bloody diarrhoea	Stem bark	Tanzania	[45]
Palpitations	Bark and leaves	Mauritius	[47]
Convulsions	Stem bark	Tanzania	[45]
Diabetes	Bark, leaves, fruits, and roots	Madagascar, Mauritius, and Sudan	[14, 47–50]
Dysentery	Bark and leaves	Mauritius	[47]
Gonorrhoea	Stem bark	Tanzania	[45]
Hepatitis	Roots and root bark	Tanzania	[45, 51]
Hernia	Stem bark	Tanzania	[45]
Hypertension	Fruits	Sudan	[50]
Intestinal worms	Roots and root bark	Eritrea and Tanzania	[8, 51, 52]
Kidney problems	Fruits	Sudan	[50]
Malaria	Bark, roots, and stem bark	Kenya and Tanzania	[8, 45, 51, 53–57]
Mouth infections	Roots	Kenya	[58]
Mouthwash	Leaf decoction taken orally mixed with leaves of *Jatropha curcas*, *Toddalia asiatica* (L.) Lam., and *Sporobolus africanus* (Poir.) Robyns & Tournay	Mauritius	[48]
Nausea	Bark and leaves	Mauritius	[47]
Oedema	Stem bark	Tanzania	[45]
Purgative	Roots	Eritrea	[52]
Stomach problems	Roots	Kenya	[58]
Tumour	Seeds	Sudan	[59]

**Table 5:** Nutritional and phytochemical composition of *Vangueria madagascariensis*.

Compound	Value	Method of compound analysis	Plant part	References
Carbohydrates (%)	14.6		Seeds	[16]
Fibre (%)	14.0 ± 0.2		Seeds	[16]
Moisture (%)	6.4 ± 0.1		Seeds	[16]
Protein (%)	22.2 ± 0.3		Seeds	[16]
Total flavonoid content (mg RE/g fresh weight)	6.7–9.0	—	Leaves, fruits, and seeds	[13]
Total phenolic content (mg GAE/g fresh weight)	35.0–122.2	—	Leaves, fruits, and seeds	[13]
Total proanthocyanidins (mg CE/g fresh weight)	42.5–185.7	—	Leaves, fruits, and seeds	[13]

*Vitamin E*				
*α*-Tocopherol (mg/100 g)	28.5–31.6	GC/MS and HPLC	Kernel oil	[16]
*β*-Tocopherol (mg/100 g)	63.8–65.7	GC/MS and HPLC	Kernel oil	[16]
*γ*-Tocopherol (mg/100 g)	4.7–5.1	GC/MS and HPLC	Kernel oil	[16]
*δ*-Tocopherol (mg/100 g)	8.4–10.5	GC/MS and HPLC	Kernel oil	[16]

*Alcohol*				
Cetyl alcohol	—	NMR	Leaves and stem bark	[60]

*Cyclitol*				
Ethyl-1-O-glucosyl-4-O-(E) caffeoyl quinate	—	NMR	Leaves and stem bark	[60]

*Flavonoid*				
Kaempferol-3-O-rhamnoside-7-O-rutinoside	—	NMR	Leaves and stem bark	[60]

*Coumarin*				
Esculetin	—	NMR	Leaves and stem bark	[60]

*Phenolics*				
Chlorogenic acid (mg/100 g)	1.0–1.2	HPLC-DAD and MS	Leaves and seeds	[17]
Ferulic acid (mg/100 g)	0.03–0.06	HPLC-DAD and MS	Leaves and seeds	[17]
Gallic acid (mg/100 g)	0.004–0.06	HPLC-DAD and MS	Bark, leaves, and seeds	[17]
Hydroxybenzoic acid (mg/100 g)	0.03–0.05	HPLC-DAD and MS	Leaves and seeds	[17]
p-Coumaric acid (mg/100 g)	0.005–0.03	GC, HPLC-DAD, MS, and NMR	Leaves, seeds, stems, and stem bark	[17, 60]
Protocatechuic acid	—	NMR	Leaves and stem bark	[60]
Scopoletin	—	NMR	Leaves and stem bark	[60]
Syringic acid (mg/100 g)	0.007–0.21	HPLC-DAD and MS	Bark, leaves, and seeds	[17]
Vanillic acid	—	NMR	Leaves and stem bark	[60]
Vanillin (mg/100 g)	0.02–0.05	HPLC-DAD and MS	Bark, leaves, and seeds	[17]

*Monomethyl ester*				
4,4-Dimethyl pimelate (%)	0.1	GC/MS	Leaves and stems	[60]
Methyl margarate (%)	1.1	GC/MS	Leaves and stems	[60]
Methyl myristate (%)	3.1	GC/MS	Leaves and stems	[60]
Methyl palmitate (%)	44.7	GC/MS	Leaves and stems	[60]
Methyl stearate (%)	10.5	GC/MS	Leaves and stems	[60]
Pentadecyl cyclohexanecarboxylate (%)	2.2	GC/MS	Leaves and stems	[60]

*Fatty acids*				
9-Hexadecenoic acid (%)	0.4	GC/MS	Leaves and stems	[60]
9-Dodecenoic acid (%)	0.2	GC/MS	Leaves and stems	[60]
8,11-Octadecadienoic acid (%)	8.9	GC/MS	Leaves and stems	[60]
9,12,15-Octadecatrienoic acid (%)	12.1	GC/MS	Leaves and stems	[60]
11-Octadecenoic acid (%)	0.1	GC/MS	Leaves and stems	[60]
Arachidic acid (%)	2.2–5.9	GC, GC/MS, and HPLC	Kernel oil and leaves	[16, 61]
Capric acid (%)	3.7–4.1	GC, GC/MS, and HPLC	Kernel oil	[16]
Docosanoic acid (%)	2.7	GC/MS	Leaves and stems	[60]
Dodecanoic acid (%)	0.2	GC/MS	Leaves and stems	[60]
Eicosanoic acid (%)	6.0	GC/MS	Leaves and stems	[60]
Erucic acid (%)	0.2–0.7	GC/MS and HPLC	Kernel oil	[16]
Heneicosanoic acid (%)	0.9	GC/MS	Leaves and stems	[60]
Hexadecadienoic acid (%)	0.5	GC	Leaves	[61]
Hexadecatrienoic acid (%)	1.3	GC	Leaves	[61]
Linolenic acid (%)	0.4–43.7	GC and GC/MS	Leaves and stems	[60, 61]
Linoleic acid (%)	0.3–63.4	GC, GC/MS, and HPLC	Kernel oil, leaves, and stems	[16, 60, 61]
α-Linoleic acid (%)	0.4–0.7	GC/MS, HPLC, and GC	Kernel oil	[16]
Myristic acid (%)	0.9–2.1	GC/MS, HPLC, and GC	Kernel oil and leaves	[16, 61]
Nonanedioic acid (%)	0.1	GC/MS	Leaves and stems	[60]
Nonadecanoic acid (%)	0.6	GC/MS	Leaves and stems	[60]
Oleic acid (%)	3.8–10.5	GC, GC/MS, and HPLC	Kernel oil and leaves	[16, 61]
Palmitic acid (%)	9.7–20.9	GC, GC/MS, HPLC, and NMR	Kernel oil, leaves, and stem bark	[16, 60, 61]
Palmitoleic acid (%)	1.0	GC	Leaves	[61]
Pentadecanoic acid (%)	0.1	GC/MS	Leaves and stems	[60]
Pentacosanoic acid (%)	0.2	GC/MS	Leaves and stems	[60]
Stearic acid (%)	5.1–9.4	GC, GC/MS, and HPLC	Kernel oil and leaves	[16, 61]
Tetracosanoic acid (%)	1.6	GC/MS	Leaves and stems	[60]
Tricosanoic acid (%)	1.0	GC/MS	Leaves and stems	[60]
*trans*-Hexadecenoic acid (%)	1.7	GC	Leaves	[61]

*Sterols*				
Campesterol (%)	22.7	GC/MS	Kernel oil	[16]
*β*-Sitosterol (%)	45.2	GC/MS and NMR	Kernel oil, leaves, and stem bark	[16, 60]
*β*-Sitosterol acetate	—	NMR	Leaves and stem bark	[60]
*β*-Sitosterol-5-*β-*O-glucosapranoside	—	NMR	Leaves and stem bark	[60]
∆-5-avenasterol (%)	1.4	GC/MS	Kernel oil	[16]
Lanosterin (%)	5.6	GC/MS	Kernel oil	[16]
Cycloartenol (%)	4.3	GC/MS	Kernel oil	[16]
(+)-24-Dammarene-3*β*-20S-diol (%)	0.7	GC/MS	Kernel oil	[16]
Stigmasterol (%)	20.1	GC/MS and NMR	Kernel oil, leaves, and stem bark	[16, 60]

**Table 6:** Summary of biological properties of *Vangueria madagascariensis* extracts.

Property assessed	Extract	Plant part	Model	Biological effects	Reference
Antibacterial	Decoction extracts	Ripe fruit	Disc diffusion	Active against *Staphylococcus aureus* with 10.7 ± 1.2 mm zone of inhibition	[13]
		Unripe fruit	Disc diffusion	Active against *Escherichia coli* with 12.7 ± 0.6 mm zone of inhibition	[13]
	Methanol	Leaf	Disc diffusion	Active against *Escherichia coli* and *Staphylococcus aureus* with 10.0 ± 2.0 mm and 11.7 ± 1.5 mm zones of inhibition, respectively	[13]
		Seed	Disc diffusion	Active against *Staphylococcus aureus* with 8.3 ± 1.5 mm zone of inhibition	[13]
	Decoction extracts	Ripe fruit	Microtitre dilution broth method	Active against *Staphylococcus aureus* with an MIC value of 12.5 mg/mL	[13]
		Unripe fruit	Microtitre dilution broth method	Active against *Escherichia coli* with an MIC value of 25.0 mg/mL	[13]
	Methanol	Leaf	Microtitre dilution broth method	Active against *Escherichia coli* and *Staphylococcus aureus* with MIC values of 6.3 mg/mL and 12.5 mg/mL, respectively	[13]
		Seed	Microtitre dilution broth method	Active against *Staphylococcus aureus* with an MIC value of 25.0 mg/mL	[13]
	Decoction extracts	Fruit	Microtitre dilution broth method	Active against *Streptococcus* group A with an MIC value of <0.1 mg/mL, *Escherichia coli* and *Streptococcus* group B (0.78 mg/mL), *Acinetobacter* spp., *Proteus* spp., and *Staphylococcus aureus* (1.6 mg/mL), *Enterococcus faecalis* and methicillin-resistant *Staphylococcus aureus* (MRSA) (3.1 mg/mL), and *Klebsiella* spp. (12.5 mg/mL)	[72]
		Leaf	Microtitre dilution broth method	Active against *Streptococcus* group A and *Streptococcus* group B with an MIC value of 0.8 mg/mL, *Enterococcus faecalis*, *Escherichia coli*, *Klebsiella* spp., and *Staphylococcus aureus* (3.1 mg/mL), and *Proteus* spp. and methicillin-resistant *Staphylococcus aureus* (MRSA) (6.3 mg/mL)	[72]
	Methanol	Fruit	Microtitre dilution broth method	Active against *Streptococcus* group A with an MIC value of 0.8 mg/mL, *Streptococcus* group B (1.6 mg/mL), *Acinetobacter* spp., *Enterococcus faecalis*, *Proteus* spp., and *Staphylococcus aureus* and methicillin-resistant *Staphylococcus aureus* (MRSA) (3.1 mg/mL), *Escherichia coli* (6.3 mg/mL), and *Klebsiella* spp. (12.5 mg/mL)	[72]
		Leaf	Microtitre dilution broth method	Active against *Enterococcus faecalis*, *Streptococcus* group A and *Streptococcus* group B with an MIC value of <0.2 mg/mL, *Escherichia coli*, *Klebsiella* spp., *Proteus* spp., and *Staphylococcus aureus* (3.1 mg/mL), and methicillin-resistant *Staphylococcus aureus* (MRSA) (6.3 mg/mL)	[72]
	Aqueous	Leaf	Agar well diffusion method	Active against *Staphylococcus aureus* with 6 mm zone of inhibition, *Bacillus cereus* (8 mm), *Escherichia coli* (10 mm), and *Klebsiella pneumoniae* (13 mm)	[60]
	Dichloromethane	Leaf	Agar well diffusion method	Active against *Staphylococcus aureus* with 6 mm zone of inhibition, *Escherichia coli* (9 mm), *Pseudomonas aeruginosa* (10 mm), *Bacillus cereus* (11 mm), and *Klebsiella pneumoniae* (15 mm)	[60]
	Ethanol	Leaf	Agar well diffusion method	Active against *Staphylococcus aureus* with 4 mm zone of inhibition, *Micrococcus luteus* (7 mm), *Bacillus cereus* (8 mm), *Escherichia coli* (10 mm), *Pseudomonas aeruginosa* (11 mm), and *Klebsiella pneumoniae* (17 mm)	[60]
	*n*-Butanol	Leaf	Agar well diffusion method	Active against *Staphylococcus aureus* with 5 mm zone of inhibition, *Escherichia coli* (10 mm), and *Klebsiella pneumoniae* (14 mm)	[60]
	*n*-Hexane	Leaf	Agar well diffusion method	Active against *Staphylococcus aureus* with 6 mm zone of inhibition, *Micrococcus luteus* (7 mm), *Bacillus cereus* (8 mm), *Escherichia coli* (9 mm), and *Klebsiella pneumoniae* (12 mm)	[60]
	Aqueous	Stem bark	Agar well diffusion method	Active against *Micrococcus luteus* with 8 mm zone of inhibition, *Bacillus cereus* with *Klebsiella pneumoniae* (9 mm), *Pseudomonas aeruginosa* (10 mm), and *Escherichia coli* (11 mm)	[60]
	Dichloromethane	Stem bark	Agar well diffusion method	Active against *Micrococcus luteus* with 8 mm zone of inhibition, *Pseudomonas aeruginosa* (9 mm), *Escherichia coli* and *Staphylococcus aureus* (10 mm), *Bacillus cereus* (12 mm), and *Klebsiella pneumoniae* (13 mm)	[60]
	Ethanol	Stem bark	Agar well diffusion method	Active against *Micrococcus luteus* with 8 mm zone of inhibition, *Escherichia coli* (10 mm), *Bacillus cereus* and *Pseudomonas aeruginosa* (11 mm), *Staphylococcus aureus* (14 mm), and *Klebsiella pneumoniae* (15 mm)	[60]
	*n*-Hexane	Stem bark	Agar well diffusion method	Active against *Staphylococcus aureus* with 9 mm zone of inhibition, *Bacillus cereus* (10 mm), and *Klebsiella pneumoniae* (12 mm)	[60]
	Aqueous	Leaf	Agar well diffusion method	Active against *Escherichia coli* with an MIC value of 15 *µ*g/ml, *Klebsiella pneumoniae* (30 *µ*g/ml), *Staphylococcus aureus* (40 *µ*g/ml), and *Bacillus cereus* (75 *µ*g/ml)	[60]
	Dichloromethane	Leaf	Agar well diffusion method	Active against *Klebsiella pneumoniae* with an MIC value of 20 *µ*g/ml, *Escherichia coli* and *Staphylococcus aureus* (25 *µ*g/ml), *Bacillus cereus* (35 *µ*g/ml), and *Pseudomonas aeruginosa* (50 *µ*g/ml)	[60]
	Ethanol	Leaf	Agar well diffusion method	Active against *Klebsiella pneumoniae* with an MIC value of 15 *µ*g/ml, *Staphylococcus aureus* (40 *µ*g/ml), *Bacillus cereus* and *Escherichia coli* (50 *µ*g/ml), and *Micrococcus luteus* and *Pseudomonas aeruginosa* (55 *µ*g/ml)	[60]
	*n*-Butanol	Leaf	Agar well diffusion method	Active against *Klebsiella pneumoniae* with an MIC value of 25 *µ*g/ml, *Staphylococcus aureus* (36 *µ*g/ml), and *Escherichia coli* (75 *µ*g/ml)	[60]
	*n*-Hexane	Leaf	Agar well diffusion method	Active against *Bacillus cereus* with an MIC value of 6.3 *µ*g/ml, *Klebsiella pneumoniae* (25 *µ*g/ml), *Staphylococcus aureus* (37 *µ*g/ml), *Escherichia coli* (60 *µ*g/ml), and *Micrococcus luteus* (75 *µ*g/ml)	[60]
	Aqueous	Stem bark	Agar well diffusion method	Active against *Pseudomonas aeruginosa* with an MIC value of 25 *µ*g/ml, *Escherichia coli* and *Klebsiella pneumoniae* (35 *µ*g/ml), and *Bacillus cereus* and *Micrococcus luteus* (75 *µ*g/ml)	[60]
	Dichloromethane	Stem bark	Agar well diffusion method	Active against *Escherichia coli* with an MIC value of 20 *µ*g/ml, *Klebsiella pneumoniae* (24 *µ*g/ml), *Pseudomonas aeruginosa* (25 *µ*g/ml), *Staphylococcus aureus* (33 *µ*g/ml), *Micrococcus luteus* (50 *µ*g/ml), and *Bacillus cereus* (55 *µ*g/ml)	[60]
	Ethanol	Stem bark	Agar well diffusion method	Active against *Klebsiella pneumoniae* and *Staphylococcus aureus* with an MIC value of 20 *µ*g/ml, *Escherichia coli, Micrococcus luteus*, and *Pseudomonas aeruginosa* (25 *µ*g/ml), and *Bacillus cereus* (50 *µ*g/ml)	[60]
	*n*-Hexane	Stem bark	Agar well diffusion method	Active against *Klebsiella pneumoniae* with an MIC value of 24 *µ*g/ml, *Staphylococcus aureus* (35 *µ*g/ml), and *Bacillus cereus* (65 *µ*g/ml)	[60]

Antidiabetic	Decoction extract	Fruit, leaf, and seed	*α*-Amylase	Decoctions active with IC_50_ values of 1.1 mg/mL (leaf), 5.3 mg/mL (unripe fruit), 6.8 mg/mL (seed), and 29.6 mg/mL (ripe fruit)	[13]
		Fruit and leaf	*α*-Glucosidase	Decoctions active with IC_50_ values of 0.5 mg/mL (unripe fruit), 0.6 mg/mL (leaf), and 15.7 mg/mL (ripe fruit)	[13]
	Methanol	Fruit, leaf, and seed	*α*-Amylase	Extracts active with IC_50_ values of 1.2 mg/mL (unripe fruit), 1.7 mg/mL (leaf), 3.8 mg/mL (seed), and 7.7 mg/mL (ripe fruit)	[13]
		Fruit, leaf, and seed	*α*-Glucosidase	Extracts active with IC_50_ values of 0.4 mg/mL (unripe fruit), 3.3 mg/mL (ripe fruit), 6.2 mg/mL (leaf), and 46.3 mg/mL (seed)	[13]
	Ethanol	Bark	*α*-Amylase	Extracts active with IC_50_ values of 11.6 *µ*g/mL	[73]
		Bark	*α*-Glucosidase	Extracts active with IC_50_ values of 1.8 *µ*g/mL	[73]

Antifungal	Several extracts	Leaf	Agar well diffusion method	*n*-Butanol and *n*-hexane active against *Candida albicans* with 9 mm zone of inhibition, ethanol (10 mm), aqueous (14 mm), and dichloromethane (20 mm)	[60]
		Stem bark	Agar well diffusion method	Aqueous and *n*-hexane active against *Candida albicans* with 14 mm zone of inhibition, ethanol (18 mm), and dichloromethane (21 mm)	[60]
		Leaf	Agar well diffusion method	Ethanol extract active against *Candida albicans* with an MIC value of 13 *µ*g/ml, dichloromethane (14 *µ*g/ml), aqueous (18 *µ*g/ml), *n*-butanol (30 *µ*g/ml), and *n*-hexane (35 *µ*g/ml)	[60]
		Stem bark	Agar well diffusion method	Ethanol extract active against *Candida albicans* with an MIC value of 15 *µ*g/ml, dichloromethane (18 *µ*g/ml), *n*-hexane (30 *µ*g/ml), and aqueous (55 *µ*g/ml)	[60]
	Chloroform	Fruit	Agar well diffusion method	Chloroform extract active against *Aspergillus niger* and *Candida albicans* with 15 mm and 14 mm zones of inhibition, respectively	[74]

Antioxidant	Decoction	Fruit, leaf, and seed	DPPH	Extracts active with IC_50_ values of 132.8 *µ*g/mL (leaf), 602.5 *µ*g/mL (ripe fruit), and 612.5 *µ*g/mL (unripe fruit and seed)	[13]
	Methanol	Fruit, leaf, and seed	DPPH	Extracts active with IC_50_ values of 9.0 *µ*g/mL (leaf), 10.0 *µ*g/mL (unripe fruit), 48.5 *µ*g/mL (ripe fruit), and 105.9 *µ*g/mL (seed)	[13]
	Decoction	Fruit, leaf, and seed	FRAP	Exhibited antioxidant activity with 319.2 (seed), 322.9 (ripe fruit), 330.8 (unripe fruit), and 350.4 (leaf) mM Trolox equivalent (TE)/g fresh weight	[13]
	Methanol	Fruit, leaf, and seed	FRAP	Exhibited antioxidant activity with 346.7 (seed), 357.1 (ripe fruit), 361.3 (unripe fruit), and 372.5 (leaf) mM Trolox equivalent (TE)/g fresh weight	[13]
	Decoction extracts	Fruit, leaf, and seed	HOCl	Active with IC50 values of 235.6 *µ*g/mL (leaf), 275.3 *µ*g/mL (unripe fruit), 982.4 *µ*g/mL (ripe fruit), and 6656.4 *µ*g/mL (seed)	[13]
	Methanol	Fruit, leaf, and seed	HOCl	Active with IC_50_ values of 223.0 *µ*g/mL (unripe fruit), 382.1 *µ*g/mL (leaf), 418.9 *µ*g/mL (ripe fruit), and 941.5 *µ*g/mL (seed)	[13]
	Decoction extracts	Fruit, leaf, and seed	OH	Active with IC_50_ values of 157.2 *µ*g/mL (unripe fruit), 261.0 *µ*g/mL (ripe fruit), 289.0 *µ*g/mL (leaf), and 803.8 *µ*g/mL (seed)	[13]
	Methanol	Fruit, leaf, and seed	OH	Active with IC_50_ values of 0.1 *µ*g/mL (leaf), 0.3 *µ*g/mL (ripe and unripe fruits), and 22.4 *µ*g/mL (seed)	[13]

Antioxidant	Decoction extracts	Fruit, leaf, and seed	NO	Active with IC_50_ values of 241.2 *µ*g/mL (leaf), 436.2 *µ*g/mL (unripe fruit), 2367.4 *µ*g/mL (ripe fruit), and 6092.4 *µ*g/mL (seed)	[13]
	Methanol	Fruit, leaf, and seed	NO	Active with IC_50_ values of 43.2 *µ*g/mL (leaf), 91.4 *µ*g/mL (unripe fruit), 219.1 *µ*g/mL (ripe fruit), and 1103.2 *µ*g/mL (seed)	[13]
	Decoction extracts	Fruit, leaf, and seed	Iron chelation	Active with IC_50_ values of 0.3 *µ*g/mL (seed), 0.6 *µ*g/mL (ripe fruit), 1.0 *µ*g/mL (unripe fruit), and 2.5 *µ*g/mL (leaf)	[13]
	Methanol	Fruit, leaf, and seed	Iron chelation	Active with IC_50_ values of 0.0009 *µ*g/mL (seed), 0.002 *µ*g/mL (leaf), 0.06 *µ*g/mL (ripe fruit), and 0.07 *µ*g/mL (unripe fruit)	[13]
	Methanol	Bark, leaf, and seed	DPPH	Extracts active with IC_50_ values of 7.8 *µ*g/ml (leaf), 31.3 *µ*g/ml (seed), and 62.5 *µ*g/ml (bark)	[17]
	Methanol	Bark, leaf, and seed	ORAC	Extracts active with 44.9 *µ*M of Trolox (seed), 47.1 *µ*M of Trolox (bark), and 72.7 *µ*M of Trolox (leaf)	[17]

Antiplasmodial	Methanol	Leaf	G-^3^H hypoxanthine	Active against *Plasmodium falciparum* with IC_50_ values of 13.4 *µ*g/ml and 34.0 *µ*g/ml against D6 and W2 strains, respectively	[55, 56]

Cytotoxicity	Crude extracts	Stem bark	MTT assay	Extracts active with EC_50_ values of 22.8 *µ*g/ml (MCF-7), 28.4 *µ*g/ml (HepG2), 34.4 *µ*g/ml (PC-3), 42.5 *µ*g/ml (A549), 44.5 *µ*g/ml (WRL-68), 53.2 *µ*g/ml (HT-29), and 64.7 *µ*g/ml (WI-38T)	[17]

Toxicity	Ethanol	Leaf and stem bark	In vivo animal toxicity activities	All extracts appear to be nontoxic with LD_50_ values of 3.8 g/kg	[60]
